# Factors determining household-level food insecurity during COVID-19 epidemic: a case of Wuhan, China

**DOI:** 10.29219/fnr.v65.5501

**Published:** 2021-03-08

**Authors:** Yu Zhang, Kui Yang, Song Hou, Taiyang Zhong, Jonathan Crush

**Affiliations:** 1 School of Geography and Ocean Science, Nanjing University, Nanjing, China; 2 School of Economics and Business Administration, Central China Normal University, Wuhan, China; 3 Balsillie School of International Affairs, Canada; Wilfrid Laurier University, Canada; University of the Western Cape, Cape Town, South Africa

**Keywords:** food insecurity, food access, HFIAS, pandemic, COVID-19, group purchase

## Abstract

**Background:**

In coping with the coronavirus disease 2019 (COVID-19) epidemic, cities adopted social isolation and lockdown measures; however, little is known about the impacts of these restrictions on household food security.

**Objective:**

This study provides a timely assessment of household food insecurity (HFI) in the Chinese city of Wuhan during the COVID-19 epidemic period and also investigates its determinant factors.

**Design:**

We collected valid data on food insecurity from 653 households in Wuhan via an online questionnaire in March 2020. The Household Food Insecurity Access Scale Score (HFIASS) was used to measure HFI, and a multiple linear regression model was used to determine the HFIASS.

**Results:**

The mean HFIASS in Wuhan was 9.42 (standard deviation: 5.82), with more than 50% of the households had an HFIASS < 9. Compared with normal conditions, lockdown measures had a huge negative impact on household food security. The results revealed that socio-demographic characteristics remained the underlying determinants of HFIASS during the epidemic. Households in Wuhan with local Hukou (city household registration) and self-owned property had a lower risk of food insecurity.

**Discussion and conclusion:**

After the restriction of conventional food access channels, intermediary food purchase methods such as group purchasing, shopping with the help of neighborhood committees, property management agents, and volunteers became the most important or the only channel for residents to access food. There were similarities in the use of these intermediary channels. Based on the probability that the epidemic will continue and the probability of similar public health-related outbreaks in the future, the study calls for a more resilient and responsive sustainable food supply system by harnessing the capacity of communities, e-commerce and rapid logistics.

## Popular scientific summary

- Household food insecurity in Wuhan was investigated at the time of the COVID-19 epidemic.
- Lockdown measures posed notable impacts to households’ food security in Wuhan.
- Households with Hukou (city household registration) and home ownership in Wuhan had a higher food security, whilst households that experienced negative income shocks in the recent past and were blocked for longer periods had a lower food security.
- The intermediary food purchasing methods based on urban logistics played an important role in household food security during the epidemic.

*Food is God’* is a saying widely used in China, indicating the vital role of food in the daily life of Chinese people. On 23 January 2020, in coping with the spread of coronavirus disease 2019 (COVID-19), Wuhan, China, was the first city to adopt a lockdown measure (1). Restrictions on social activities and mobility led to a sharp rise in uncertainty over food security for more than 9 million residents who remained in the city (2, 3). The lockdown policy had two main impacts on people’s daily food access (4): on the demand side, the closure of the city led to panic buying in the short term (5), while the unemployment and insufficient income caused by the continuous closure of the city reduced people’s ability to pay, and on the supply side, conventional food outlets were forced to close, logistics were disrupted and food prices rose. Since April 1, more than 40 countries, including Italy and some parts of the United States, have implemented local closures and evacuation measures similar to those in China (3), and food security in restricted environments has become a global issue. While there is no doubt that the epidemic has had an impact on food security, little is known about how food security at the household level has changed as a result of the epidemic, especially in an epidemic epicenter like Wuhan, China.

‘Food security exists when all people, at all times, have physical and economic access to sufficient, safe and nutritious food to meet their dietary needs and food preferences for an active and healthy life’ (6). This means that household food insecurity (HFI) occurs when any member of the household is unable to have an active and healthy lifestyle because of food issues (7). Food security is usually explained in terms of four dimensions: availability in quantity; access in economic, logistic, and socio-cultural; utilization in high quality and safety; and stability at all times (8). Thus, food security is not only about food shortages and hunger but also about the broader issues of health and balanced diets (9). In terms of influencing factors, HFI is widely believed to be associated with social and demographic characteristics, such as gender, family location, income, main source of income, housing, education and household structure (10–14). As a comprehensive proxy for many factors, income (or poverty) is considered to be the strongest and most consistent variable affecting food security (6, 9). Household food security is not a static concept; recent negative income shocks (NIS), migration, and increases or decreases in household size have all increased the probability of food insecurity (15). At the broader social level and in particular regions, household food security is associated with more complex factors, such as environmental stresses, regional conflicts, floods or earthquakes, and the collapse of AIDS-related social capital (16–18).

When HFI occurs, social relief plays a role (15). Experience in the United States showed that the Supplemental Nutrition Assistance Program’s benefits reduced the probability of being food insecure by roughly 30% and reduced the probability of being very food insecure by 20% (19). But emergency relief services usually do not address the root causes of food insecurity (9). The above studies have promoted the research on food insecurity, which has important empirical significance. However, most of the relevant studies are based on daily background or regional and temporary shocks, when facing global public health events there is still a lack of understanding on how people’s food security changes and how food security is implemented.

COVID-19 is extremely infectious disease (20), and the World Health Organization (WHO) declared a pandemic on 11 March 2020 (21), indicating that COVID-19 would bring widespread global impact and long-term uncertainty. Together with the increasing complexity of the global socio-political ecology, how household food security is changing in these contexts is a scientific question that requires urgent research.

Understanding the household food security situation during the COVID-19 pandemic can help facilitate relief measures, which can also contribute to promoting the construction of sustainable cities and communities. As the epicenter of the outbreak in China, Wuhan was selected for this study. Through a rapid online survey, the aims of this study were to 1) characterize food insecurity in households during the epidemic in Wuhan, and 2) quantify social and economic factors and the relationship between demographic characteristics and HFI. Based on the possibility of the continuation of the epidemic and the probability of the outbreak of similar public health events in the future, the study of food security at the household level during the epidemic in Wuhan can provide valuable empirical reports for building sustainable food security mechanisms.

## Data sources and study area

### Study area

**Fig. 1:**
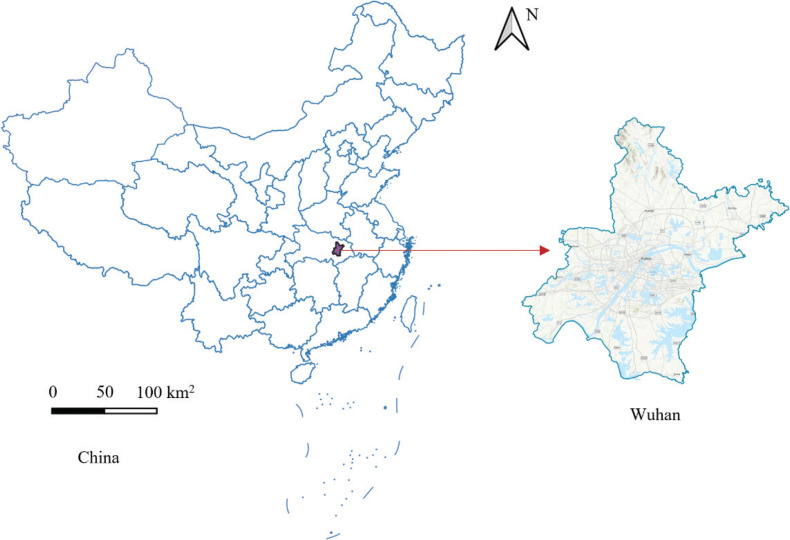
Location of Wuhan, China.

As the city where the COVID-19 epidemic was first reported and was most severe in China, Wuhan was selected as the study area. Located in the heart of central China and the capital city of Hubei Province (Fig. 1), Wuhan has a population of 11.21 million (of which 9.06 million are registered residents) with 13 administrative districts (22, 23).

In December 2019, Wuhan reported the emergence of COVID-19 infections (24), and on 23 January 2020, Wuhan closed its public transportation, airports, and railway stations (1). Subsequently, a series of regulatory measures were developed; gatherings were banned; entertainment venues, schools and factories were closed; residents were restricted in their mobility; and operations of wet markets and supermarkets were disrupted (25). On 11 February 2020, residential areas were placed under lockdown management in Wuhan (26). By 24 March 2020, Wuhan had reported 50,006 confirmed cases of COVID-19 (27).

### Data collection

This study is based on data obtained from a household food consumption survey conducted in Wuhan in March 2020. We designed a Chinese online interview questionnaire titled The Impact of COVID-19 on Household Food Consumption, which was composed of six parts: 1) social demographic characteristics of interviewees and their families, 2) the modified Household Food Insecurity Access Scale (HFIAS), 3) which types of food had been affected by the epidemic, 4) what were the main food access channels for families during the closure of the city, 5) infection status in the community and protective expenses of the interviewee’s household, and 6) brief essay questions, such as ‘what do you think are the most effective ways and methods to purchase food since the COVID-19 epidemic’? The second part (HFIAS) is a widely used indicator system developed by Food and Nutrition Technology Assistance (FANTA) to measure HFI (28). The original language of the HFIAS module was English, and the questions in the online survey were translated into Chinese. Although this study did not directly measure the nutritional status of each household through calorie consumption methods, previous studies in multiple regions have shown that HFIAS is a convenient and effective method with good internal consistency and reliability levels (29, 30).

**Fig. 2:**
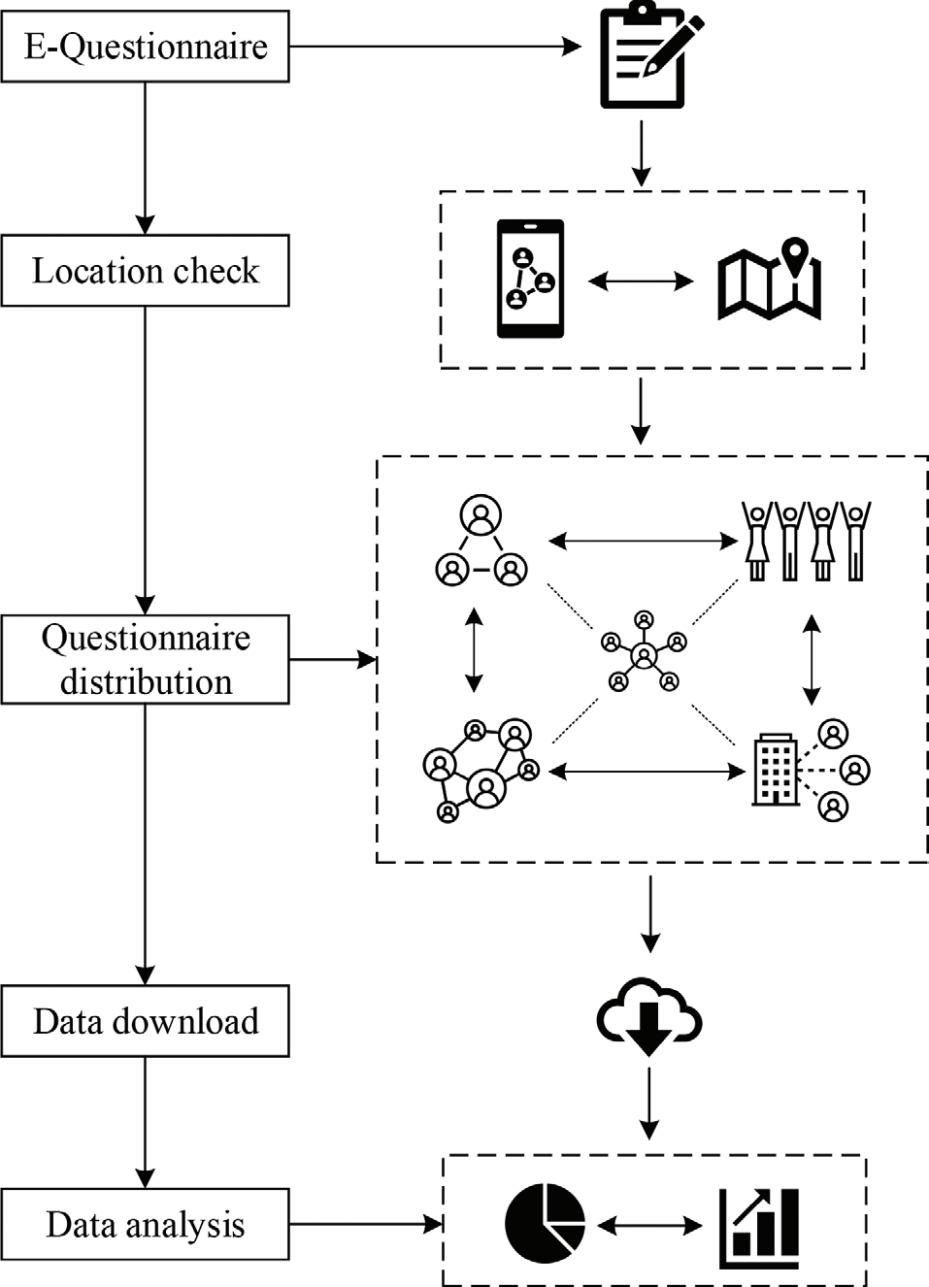
Flow chart of data collection.

The questionnaire was based on Wenjuan Xing (Ranxing Information Technology Co., LTD. Changsha, China), a popular e-questionnaire platform in China. Figure 2 shows the flow chart of data collection. After entering questions on the website, researchers can get access to a link to the questionnaire and a QR (Quick Response) code. The questionnaire was distributed through WeChat (Tencent Inc., Shenzhen, China), the most widely used social media in China (similar to Facebook internationally). There were many WeChat groups and questionnaires were distributed across those interconnected community networks. We set restrictions on access to questionnaire in order to ensure the effectiveness of information collection. Only the IP (Internet Protocol) address of connected devices in Wuhan could access and complete the questionnaire. At the beginning of the questionnaire, there was a reminder that ‘Each Household Only Needs to Fill in One Copy’. A total of 918 responses were obtained from March 25 to March 31, 2020, screening out responses that were not carefully answered and missing key data resulted in a total of 653 households’ valid data.

## Methods

### The Modified Household Food Insecurity Access Scale

The modified HFIAS measured HFI by the frequency of nine food insecurity incidents. Options to each incident include ‘never’, ‘Rarely (1–2 times)’, ‘Sometimes (3–10 times)’, and ‘Often or Always (more than 10 times)’. The options assigned a score of 0–3, and the total HFIASS was in the range of 0–27, with higher scores indicating a higher food insecurity.

### Dependent and independent variables

Table 1 lists the variables used in this study along with their definition, expected sign and key statistical information. HFIASS is the dependent variable. The main factors considered to potentially contribute to HFI are as follows:

1. *Housing property*: The property status of the house has been linked to food insecurity in previous research (10, 31). This study considered both housing purchased by households and housing allocated to individuals during China’s planned economy period to be self-owned housing, as opposed to rented housing. It is generally accepted that people who own their houses, as opposed to renters, usually have stable social connections and jobs in the local area, and in a crisis period, they have a lower risk of food insecurity. In this study, the abbreviation HP is used to refer to Housing Property, and the coefficient is assumed to be negative.
2. *Days of complete closure*: This indicates the duration of mobility restrictions in the community since 23 January 2020. In general, the longer the restrictions in mobility, the higher the level of food insecurity in the household. This study uses days of complete closure (DCC) as a proxy for this variable and assumes that its coefficient is positive.
3. *Community infection*: This indicates the presence of COVID-19-infected persons in the community. Infected persons may influence control measures in their communities, but their impact on household food access was uncertain. There are two possibilities. On one hand, when there were infected people in the community, stricter control measures and panic emotions would aggravate the food insecurity status of households; on the other hand, due to the presence of infected people in the community, more social forces may invest in the material support of the community. This study used CI to refer to this variable.
4. *Hukou*: Household Registration. A system with Chinese characteristics indicates whether a resident is a native. In China, individual Hukou is associated with many social rights and interests, such as education, medical insurance, and social security (32). It is generally believed that having the household registration in the city conveys relatively good social security support. The coefficient of the Hukou was assumed to be negative.
5. *Long-term resident*: Long-term resident status may be relevant to eligibility for temporary relief policies. In addition, long-term residents may have stronger local relationships, which can help them get food during a crisis. In the study, the long-term residents were defined as having lived in Wuhan for more than 6 months in a year. This study used LTR to refer to this variable, and its coefficient was assumed to be negative.
6. *Expenditures on medical and protective equipment*: This includes medical expenses and household expenses for equipment to protect against the virus, such as masks, disinfectants, and hand sanitizers. Although food has low consumption elasticity, there is still a certain competitive relationship between food consumption and other expenditures, and people may reduce their food consumption budget due to the increase of necessary expenditures in other areas. In this study, EMPE is used to refer to this variable, and its coefficient was assumed to be positive.
7. *Household size*: This indicates the number of household members. In a study of second-tier Chinese cities similar to Wuhan, there was a statistically significant relationship between household size and household food diversity (33). As in regional conflicts, the size of the household was related to the likelihood of receiving relief (34). During the epidemic, however, the impact of household size on HFI was uncertain. Larger household size means more demand for food consumption, which, in turn, means that households had more social relationships to access needed food resources.
8. *Pregnant or infant in household*: This indicates whether there is a pregnant woman or an infant in the household. In the international literature, food insecurity in the household with pregnant women or babies was higher (35). Typically, when there is a pregnant woman or a baby in the household, there are relatively special food and nutritional needs, such as milk powder, which may be difficult to meet under lockdown control. This study uses PIH to refer to the presence of a pregnant woman or infant in the household and assumed a positive coefficient.
9. *Negative income shocks*: This indicates whether the epidemic has had a negative income impact on households. The recent NIS experienced by households had been proved to have a negative impact on household food security (15), and therefore, the coefficient of this variable is assumed to be positive, and is abbreviated as NIS.
10. *Ways for households to access food during the epidemic*. With the closure of the community, individuals were unable to buy food from supermarkets or markets and needed the help of an intermediary. Studies have shown that in the early days of the epidemic, both Italy and China were able to account for the nutritional needs of their populations by adopting unconventional food supply measures (36). In Wuhan, there were mainly four purchase methods: 1) group purchases based on city logistics (Group purchase, GP), 2) purchases with the help of property management agent (Property management agent purchase, PMAP), 3) purchases with the help of neighborhood committees (Neighborhood committee purchase, NCP), and 4) purchases with the help of volunteers (Volunteer purchase, VP). Each household may use more than one purchasing method, and this study looked at the individual and combined effects of these intermediary purchase methods. The total number of food purchasing methods used by the household was expressed as Total purchase method number (TPN) and was assumed to have a negative coefficient.

**Table 1:** Dependent and independent variables

Variables	Definition	Expected sign^1^	Mean	Standard deviation
Dependent variable	Household Food Insecurity Access Scale Score with values ranging from 0 to 27		9.42	5.82
Independent variables	Housing property, HP = 1 for self-owned property, 0 for otherwise	−	0.77	0.42
	Days of complete closure (days), DCC	+	53.79	11.7
	Community infection, CI = 1 for the community had confirmed cases (s), 0 for otherwise		0.52	0.50
	Hukou, 1 for Wuhan, 0 for otherwise	−	0.74	0.44
	LTR, 1 for long-term resident, 0 for otherwise	−	0.85	0.36
	Expenditures on medical and protective equipment (thousand Chinese Yuan), EMPE	+	1.83	4.45
	Dummy variable for household size, HHSL = 1 for no less than 7 persons, 0 for otherwise		0.08	0.27
	Pregnant or infant household, PIH = 1 for household with pregnant (s) or infant (s), 0 for otherwise	+	0.33	0.47
	Negative income shock, NIS = 1 for yes, 0 for no	+	0.50	0.50
	Group purchase, GP = 1 for yes, 0 for no	−	0.67	0.47
	Property management agent purchase, PMAP = 1 for yes, 0 for no	−	0.39	0.49
	Neighborhood committee purchase, NCP = 1 for yes, 0 for no	−	0.53	0.5
	Volunteer purchase, VP = 1 for yes, 0 for no	−	0.36	0.48
	TPN, total purchase method number, range from 0 to 4	−	2.02	1.06

1 The expected sign shows the relationship of this variable to Household Food Insecurity Access Scale Score (HFIASS), with ‘+’ indicating a positive correlation and ‘−’ indicating a negative correlation. For example, the sign for housing property is ‘−’, which indicates that self-owned property households will have a relatively low HFIASS.

### Statistical analysis

Firstly, independent *t*-test and variance analysis were used to visually demonstrate the differences in HFIASS among different groups with different socio-demographic characteristics. Secondly, a multiple linear regression model was established to quantify the relationship between relevant variables (independent variables in Table 1) and HFIASS.

Given the complexity of food access during an epidemic, three models were created. Model I included all food purchase methods available during the epidemic. To explore the effect of the number of household food purchasing routes on HFIASS, Model II was built and TPN was the variable for the total purchase method one household used. Model III explored the combined effect of the food purchasing patterns of household ownership, expressed as a multiplication of variables, for example, one household that used GP and PMAP at the same time will be noted as GP*PMAP.

The questionnaire data were initially processed using SPSS25 (IBM Corp., Armonk, NY, USA), and HFIASS analysis for different sociodemographic characteristic groups and multiple linear regression model were performed using Stata16 (StataCorp LP, College Station TX, USA).

## Results

### The status of HFI

Table 2 presents a statistical summary of the HFIASS of households interviewed during the epidemic in Wuhan, where more than 25% of households had an HFIASS within 5, more than 50% had an HFIASS below 9, close to 60% had an HFIASS between 6 and 15, and less than 15% had a score above 16.

**Table 2:** Frequency distribution of HFIASS for the sample in Wuhan (n = 653)

Household Food Insecurity Access Scale Score (HFIASS)	Frequency	%	Cumulative	HFIASS	Frequency	%	Cumulative
0	26	3.98	3.98				
1	13	1.99	5.97	15	18	2.76	85.3
2	24	3.68	9.65	16	11	1.68	86.98
3	40	6.13	15.77	17	10	1.53	88.51
4	30	4.59	20.37	18	19	2.91	91.42
5	38	5.82	26.19	19	14	2.14	93.57
6	43	6.58	32.77	20	11	1.68	95.25
7	53	8.12	40.89	21	9	1.38	96.63
8	49	7.5	48.39	22	3	0.46	97.09
9	49	7.5	55.9	23	2	0.31	97.4
10	57	8.73	64.62	24	3	0.46	97.86
11	40	6.13	70.75	25	2	0.31	98.16
12	24	3.68	74.43	26	6	0.92	99.08
13	28	4.29	78.71	27	6	0.92	100
14	25	3.83	82.54	Total	653	100	

Table 3 shows the cumulative number of confirmed COVID-19 cases in each district of Wuhan before the survey, as well as the regional distribution of the interviewed households and HFIASS. In Jiang’an and Jianghan districts, where the epidemic was more severe, the average HFIASS was higher, indicating that households in the area had a higher degree of food insecurity. On the whole, however, there is no significant correlation between the mean HFIASS and the cumulative number of confirmed cases in the region, and the Pearson correlation coefficient between them is only 0.18. The mean HFIASS for all households surveyed was 9.42, with a standard deviation of 5.82.

**Table 3:** Cumulative confirmed cases (until 24 March) and distribution of households interviewed (n = 653)

District	Cumulative confirmed cases	Obs	Household Food Insecurity Access Scale Score	
			Mean	Standard deviation
Jiang’an	6,549	23	11.00	7.27
Jianghan	5,183	30	10.10	6.04
Qiaokou	6,834	19	9.26	4.77
Hanyang	4,670	28	8.00	4.66
Wuchang	7,458	49	10.53	7.62
Qingshan	2,782	32	10.41	6.13
Hongshan	4,679	144	8.99	4.80
Dongxihu	2,462	15	11.27	6.09
Caidian	1,416	65	8.75	6.01
Jiangxia	848	69	9.30	5.55
Huangpi	2,114	48	9.77	6.91
Xinzhou	1,072	53	9.08	6.08
Eastlake Development Zone	2,148	36	10.44	5.07
Unkown		42	8.20	5.48
Total	48,215	653	9.42	5.82

### HFIASS among households with different socio-demographic characteristics

Table 4 demonstrates HFIASS for the different socio-demographic characteristic groups. It shows that compared with the self-owned housing group, the HFIASS of the renters is higher. When the household had Hukou registered in Wuhan, its HFIASS was lower, but with no significant differences. The HFIASS is higher for those with low and no income in the previous month. Overall, the higher the per capita household income in the previous month, the lower the HFIASS.

**Table 4:** HFIASS among households with different socio-demographic characteristics (n = 653)

Socio-demographic characteristics		*n* (%)	Household Food Insecurity Access Scale Score (mean ± standard deviation)	*P*
Housing property	Self-owned	500 (77)	9.16 ± 5.80	0.01
	Otherwise	153 (23)	10.25 ± 5.83	
Hukou	Wuhan	480 (74)	9.24 ± 5.83	0.10
	Otherwise	173 (26)	9.92 ± 5.80	
Long term resident	Yes	553 (85)	9.44 ± 5.70	0.57
	Otherwise	100 (15)	9.30 ± 6.50	
Household size	No more than 2	123 (19)	10.07 ± 6.30	0.15
	3–6	479 (73)	9.32 ±5.55	
	no less than 7	51 (8)	8.75 ± 6.14	
Household structure	Female-centered	43 (7)	9.63 ± 6.59	0.53
	Male-centered	45 (7)	9.73 ± 6.28	
	Nuclear	284 (43)	9.15 ± 5.61	
	Extended	237 (36)	9.47 ±5.63	
	Otherwise	44 (7)	10.30 ± 6.95	
Pregnant or infant household	Yes	213 (33)	9.36 ± 5.89	0.61
	Otherwise	440 (67)	9.45 ± 5.80	
Household income per capita (Chinese Yuan)	0	58 (9)	12.72 ± 6.36	0.01
	No more than 1,000	39 (6)	10.05 ± 6.94	
	1,000–3,000	79 (12)	9.09 ± 5.48	
	3,000–8,000	81 (12)	8.85 ± 5.14	
	No less than 8,000	49 (8)	10.53 ± 6.30	
	Unknown	347 (53)	8.84 ± 5.58	

Note: *P* for trend.

### Regression estimation results

The estimated results of the three models are shown in Table 5. In all the three models, the coefficients of HP, DCC, Hukou, EMPE, and NIS were significant at the 5 or 1% level, while the coefficients of other variables were not statistically significant. Housing property was strongly associated with HFI, with HFIASS for self-owned property groups 1.39–1.43 points lower than for renters. Hukou had a similar effect on housing property. If a household had Wuhan Hukou, its HFIASS would be 2.01–2.09 points lower relative to foreign Hukou. As expected, the coefficient on DCC was positive, indicating that the longer the closure time, the higher the HFIASS. EMPE and NIS both had negative effects on household food security. For every thousand Chinese Yuan increase in a household’s expenditure on medicines and protection, its HFIASS was about 0.14 points higher. For households who experienced NIS, their HFIASS were relatively high by about 2.5 points.

**Table 5:** Multiple linear regression results for HFIASS (n = 411)

Model^1^	I	II	III
Housing property	−1.424**	−1.425**	−1.392**
	(−2.33)	(−2.35)	(−2.26)
Days of complete closure	0.0498**	0.0487**	0.0495**
	(2.42)	(2.38)	(2.4)
Community infection	0.527	0.581	0.532
	(0.92)	(1.04)	(0.93)
Hukou	−2.073***	−2.017***	−2.097***
	(−3.09)	(−3.07)	(−3.09)
Long-term resident	0.38	0.317	0.472
	(0.34)	(0.29)	(0.42)
Household size	−0.931	−1.037	−0.722
	(−0.83)	(−0.93)	(−0.65)
Expenditures on medical and protective equipment	0.135**	0.142**	0.139**
	(2.38)	(2.53)	(2.39)
Negative income shock	2.532***	2.517***	2.521***
	(4.90)	(4.89)	(4.89)
Pregnant or infant household	−0.184	−0.183	−0.113
	(−0.34)	(−0.33)	(−0.21)
Group purchase (GP)	0.211		0.352
	(0.33)		(0.51)
Property management agent purchase (PMAP)	0.0654		0.255
	(0.13)		(0.46)
Neighborhood committee purchase (NCP)	−0.373		−0.343
	(−0.72)		(−0.60)
Volunteer purchase (VP)	−0.200		−0.536
	(−0.38)		(−0.90)
Total purchase method number		−0.14	
		(−0.63)	
GP*PMAP			−1.306*
			(−1.67)
GP*NCP			−0.581
			(−0.46)
GP*VP			0.814
			(1.09)
PMAP*NCP			1.362
			(0.98)
_cons	7.353***	7.620***	7.186***
	(4.30)	(4.58)	(4.14)
*N*	411	411	411
*R*^2^	0.131	0.131	0.135

1 The definitions of each variable are shown in Table 1.

Note: *t* statistics in parentheses;

* *P* < 0.1

** *P* < 0.05

*** *P* < 0.01.

All the models showed puzzling results. In Model I, all the types of food purchase methods were not significant, and in Model II, the variable TPN was still not significant. In Model III, four combinations of food purchase methods were included, and the results showed that when households had both GP and PMAP, they tended to encounter lower food insecurity (*P* = 0.10). Other combinations of food purchase method were not statistically significant. The regression results seem to indicate that the number and type of methods for food purchase have no significant effect on the food security of the household, but certain combinations of food purchase methods may have significant influence.

## Discussions

There are various forms and causes of HFI. In this study, the HFIAS module was used to measure the HFI during the epidemic in Wuhan, China, and analyze the determining factors of HFIASS. The results showed that the mean of HFIASS in Wuhan was 9.42 (standard deviation: 5.82). Compared with a similar study, which used an HFIAS module conducted in Nanjing (a Tier-2 city like Wuhan) in 2015, the mean value of HFIASS in Wuhan was much higher than that in Nanjing where the mean value was 0.61 (37). This indicates that the lockdown measure under COVID-19 epidemic had a huge negative impact on the urban food system. In contrast to some existing literature (14, 31, 34), household size’s coefficient was not statistically significant, indicating that the population-scale effects of HFI were not evident under the epidemic. In all models, the presence of infected persons in the community (CI) and the existence of PIH were not significant, suggesting that these factors were not significantly associated with household food security. Similar to international studies on food insecurity in non-epidemic conditions (11, 15, 16), socio-demographic characteristics such as NIS, HP, and Hukou remain key variables associated with HFI even during an epidemic, suggesting that these factors underlie the impact on food security. But the epidemic brought about a complete restriction of mobility, with interlinking variables and cascading layers confounding simple solutions (38), and therefore, traditional access to food failed and household food security became more complex.

### The impact of intermediary food purchasing methods on household food security

The regression results in Table 5 revealed the four methods through which Wuhan residents purchased food and the number of methods used by households during the epidemic, with Model I showing that all methods were not statistically significant and Model II showing that the number of methods used by households also had no significant effect on food insecurity. However, insignificance does not mean that these methods were ineffective or unimportant; on the contrary, it is more important to further explore the reasons for their insignificance.

We analyzed the household food purchase methods and found that each household had at least one type of intermediary food purchase method, and more than 30% of households used three or more purchase methods at the same time. Over 50% of households chose to buy food through group purchase and property management agents, with over two-thirds using group purchase. During the epidemic, in-person food purchases at wet markets or supermarkets remained the main method used across China (3); however in Wuhan, where strict controls were in place, group purchases became the main source of food for households. The above-mentioned facts reveal the reason why these factors were not significant in the regression model. At least at the HFIASS level, all the channels for purchasing foods worked simultaneously, and the differences in their effects were not significant across channels. There was also no significant difference in the number of methods used by households for HFIASS. This result is consistent with those of slightly earlier studies on household food diversity during the epidemic in China, where it showed that different methods of purchasing food did not cause differences in household food diversity (3).

**Fig. 3:**
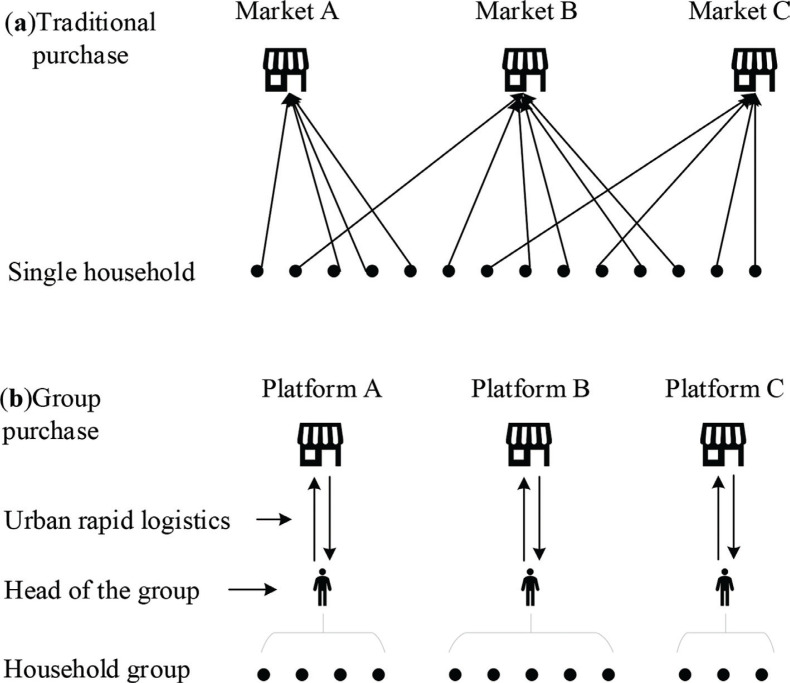
The diagrams of traditional purchase and group purchase.

On the eve of the removal of key officials in Hubei province and Wuhan, the city government adopted stricter restrictions on the daily activities of residents. From 11 February 2020, all residential communities in the city would be under closure management (26). In succession after a week, various districts in Wuhan ordered supermarkets and commercial stores to suspend opening to individuals and to only receive group purchase customers from communities, enterprises, and institutions (39). The channels for individuals or single households to independently go to supermarkets and wet markets to buy food had been blocked, and intermediate-based food purchasing became the most important or even the only food source for households. Take-out food can effectively reduce the frequency and range of contact between residents and the outside world, which can play a role during the epidemic. As of March 4, 2020, there were 41 e-commerce platforms offering online food purchasing services in Wuhan (40). Group purchase was an intermediary food access method based on takeout, but it was different from traditional takeout or online shopping to some extent. Through WeChat groups, WeChat mini programs, and other Apps, residents submitted a list of shopping needs. A leader was usually set up in each community or building, and that leader would summarize the shopping lists for the supermarket. After the supermarket completed sorting, the goods would be delivered to the leader or designated pick-up point within 1–2 days through express delivery, bus, urban errand express delivery, and other services. Compared with individual online purchases, group purchases usually required a certain order size, at which point merchants would waive shipping fees. Figure 3 illustrates the difference between traditional shopping and group purchasing. In contrast to traditional shopping, group purchasing can limit shopping during an epidemic to a few repeated contacts (41), helping to reduce the risk of infection among residents and maintaining the effectiveness of the social isolation measures.

Similar to group purchase, the use of property management agents, neighborhood committees, and volunteers for purchasing foods were all intermediary-based food access methods. However, due to the large resident base, the services provided by property companies, neighborhood committees, and volunteers were very limited, while the group purchase formed by household and supermarket self-organization was easier to set up as a service network covering the city in the decentralized system. Intermediary-based food purchasing also had some limitations. For example, customers had fewer categories to choose from, prices of items had risen and food freshness could not be guaranteed (42). Therefore, these intermediary purchase methods could not keep household food access as usual, but they played a basic role of food supply during the lockdown period.

### The role of Hukou and housing property in ensuring household food security

*‘Disease is often said to be a great leveller, striking the rich and poor alike. However, the COVID-19 pandemic has thrown into stark contrast the inequalities inherent in our food systems’* (38).

In the United States, the epidemic has widened health and nutrition disparities across income groups and races (43). A similar situation has been observed in China. Hukou and HP led to significant food security differentiation. In China, Hukou is an indication of identity, but it also serves as a criterion for policy targeting. It is often given at the time of an individual’s birth, and there are costs associated with changing Hukou. For a long time in China, residents who wanted to change their Hukou from small cities to first- or second-tier cities were often restricted in terms of education, type of work, number of years worked, and length of social security contributions (44). Hukou is linked to resources such as education, health care and social security, which are clustered in Tier-1 and Tier-2 cities, making eligibility for Hukou a highly competitive and scarce resource. Wuhan’s early relief measures focused on the local registered households, and only at a later stage the coverage was expanded from local to non-registered households. If non-registered households wanted epidemic relief, they had to take the initiative to apply. Compared with registered residents, there were certain differences in the relief measures and strength of assistance for non-registered residents (45). In terms of HP, a tenant was not the owner of the house, and therefore, did not have full access to the services of the property management agent. Relief measures during the epidemic were linked to Hukou and HP, which can easily lead to a bias in relief coverage, making the non-registered households and tenants more vulnerable to the impact of food insecurity. This is a systemic inequality that simultaneously affects the sustainability of cities and communities.

### A sustainable food security system under COVID-19 epidemic

The non-pharmacological intervention key policies to reduce COVID-19 transmission include maintaining physical distance and reducing social interaction (41, 46). Social distancing and containment measures taken in multiple countries during the epidemic have been shown to significantly reduce the number of new cases of COVID-19 (20). However, behind the lockdown there was a huge social and economic cost, which hit every household, often first affecting daily food and nutrition. In the past, food systems were designed based on routine scenarios that could be anticipated, but in the face of an epidemic, more responsive, resilient, and sustainable food supply systems are needed. After Wuhan implemented a closed community management, intermediary food purchases based on group purchasing and communities were the main methods for residents to purchase food. In the case of Wuhan, the intermediary-based food access methods covered all surveyed households. The automation of services, such as fast logistics, e-commerce, and electronic payments, has been a major goal in China (47). These facilities may not be designed for a pandemic, but they help build more resilient food security systems during an outbreak. To address the probability of a continuing epidemic, and similar public health events that may occur in the future, we need to plan for the medium to long term and promote the digital and decentralized transformation of food supply systems (38, 47).

### Research limitations

Given the risks associated with the COVID-19 epidemic and the circumstances of the city lockdown, we were unable to enter Wuhan to conduct field research. There may be some selection bias and insufficient sample size questions in the study sample. First, the study used a quick, web-based survey. Online questionnaires cannot use probability-based sampling (e.g. stratified sampling) to identify respondent households, but the sample spatially covered most areas of Wuhan, with no significant concentration or sparseness. The questionnaire for this study was distributed via WeChat, the most popular social media platform in China. In 2019, China had a total population of about 1.4 billion people, of which WeChat had more than 1.15 billion monthly active users (48). In WeChat, households in the same neighborhood would create groups based on location, and households in different neighborhoods would create groups based on other relationships, such as work relationships, so that different classes of people were connected through virtual networks. This study assumes that the pattern of questionnaire dissemination in WeChat groups was similar to a random sample or snowball sample of households in reality. In an earlier study on household food diversity during the epidemic, the authors used a similar online survey (3). Second, due to the characteristics of online interviews, marginalized groups such as the elderly living alone who did not use social software very often were not likely to be covered. It is generally accepted that older people living alone had higher levels of food insecurity during the epidemic. The omission of marginal groups may result in a lower HFIASS for the samples than the overall HFIASS. Third, similar to other research, which used online surveys (43), the answers of all the questions in the questionnaire were self-reported and may deviate from the real situation. However, the fact that the online survey was completely anonymous can alleviate some of this concern.

## Conclusions

This timely study reports on the HFIASS and its determining factors in Wuhan, a city at the center of China’s experience of the COVID-19 epidemic. During the lockdown period from January to March 2020, more than 25% of households had an HFIASS within 5, more than 50% had an HFIASS below 9, close to 60% had an HFIASS between 6 and 15, and less than 15% had a score above 16. Even in epidemic situations, socio-demographic characteristics continue to be the basis for determining the food security of households. Households who own their own housing and have local Hukou tend to have lower food insecurity and nutritional risks. NISs to households can have a negative impact on food security. When communities were closed, intermediary-based food purchases became the most important source of food access for households; however, there were no significant differences in the impact of different types of intermediary food purchase method on HFIASS.

Based on this research and the likelihood of the epidemic persisting, this study calls for a more resilient and responsive sustainable food supply system, drawing on the capacity of communities, e-commerce, and rapid logistics.

## Conflict of interest and funding

The authors declare no conflict of interest. This research was supported by an operating grant from the Canadian Novel Coronavirus (COVID-19) Rapid Research Funding Opportunity with funding from the Social Sciences and Humanities Research Council (Grant No.440234).

## References

[1] Novel Coronavirus Epidemic Prevention and Control Headquarters in Wuhan. Notice of Wuhan novel coronavirus infected pneumonitis epidemic prevention and control headquarters (No. 1). Available from: http://www.gov.cn/xinwen/2020-01/23/content_5471751.htm [cited 21 May 2020].

[2] Health Times. Where are the more than 5 million people who left Wuhan? These three places have the most! Available from: http://www.jksb.com.cn/html/2020/jjxxgzbd_0127/158668.html [cited 21 May 2020].

[3] Zhao A, Li ZY, Ke YL, Huo SS, Ma YD, Zhang YM, et al. Dietary diversity among Chinese residents during the COVID-19 outbreak and its associated factors. Nutrients 2020; 12(6): 1699. doi: 10.3390/nu12061699 PMC7352896 32517210

[4] Hobbs JE. Food supply chains during the COVID-19 pandemic. Can J Agric Econ 2020; 68(2): 171–6. doi: 10.1111/cjag.12237

[5] The Economic Observer. After the lockdown of Wuhan, citizens scrambled for supplies. Available from: https://new.qq.com/omn/20200123/20200123A0HVZQ00.html?pc [cited 21 May 2020].

[6] FAO. Rome Declaration on World Food Security and World Food Summit Plan of Action, Rome 1996. Available from: http://www.fao.org/3/w3613e/w3613e00.htm [cited 14 May 2020].

[7] Coleman-Jensen A, Rabbitt MP, Gregory CA, Singh A. Household Food Security in the United States in 2018. Available from: https://www.ers.usda.gov/webdocs/publications/94849/err-270.pdf?v=955.5 [cited 01 November 2020].

[8] Simon GA. Food security: definition, four dimensions, history. Available from: http://www.fao.org/fileadmin/templates/ERP/uni/F4D.pdf [cited 17 May 2020].

[9] Seivwright AN, Callis Z, Flatau P. Food insecurity and socioeconomic disadvantage in Australia. Int J Environ Res Public Health 2020; 17(2): 559. doi: 10.3390/ijerph17020559 PMC7014009 31952327

[10] Tarasuk V, Fafard St-Germain AA, Mitchell A. Geographic and socio-demographic predictors of household food insecurity in Canada, 2011–12. BMC Public Health 2019; 19(1): 12. doi: 10.1186/s12889-018-6344-2 30606152 PMC6318847

[11] Cordero-Ahiman OV, Vanegas JL, Beltran-Romero P, Quinde-Lituma ME. Determinants of food insecurity in rural households: the case of the Paute River Basin of Azuay Province, Ecuador. Sustainability 2020; 12(3): 946. doi: 10.3390/su12030946 PMC7923421 33672453

[12] Naja F, Hwalla N, Fossian T, Zebian D, Nasreddine L. Validity and reliability of the Arabic version of the household food insecurity access scale in rural Lebanon. Public Health Nutr 2015; 18(2): 251–8. doi: 10.1017/S1368980014000317 24702865 PMC10271416

[13] Hadley C, Lindstrom D, Tessema F, Belachew T. Gender bias in the food insecurity experience of Ethiopian adolescents. Soc Sci Med 2008; 66(2): 427–38. doi: 10.1016/j.socscimed.2007.08.025 17931763 PMC2791354

[14] Nyangasa MA, Buck C, Kelm S, Sheikh M, Hebestreit A. Exploring food access and sociodemographic correlates of food consumption and food insecurity in Zanzibari households. Int J Environ Res Public Health 2019; 16(9): 1557. doi: 10.3390/ijerph16091557 PMC6539455 31060201

[15] Swann CA. Household history, SNAP participation, and food insecurity. Food Policy 2017; 73: 1–9. doi: 10.1016/j.foodpol.2017.08.006

[16] Misselhorn AA. What drives food insecurity in southern Africa? a meta-analysis of household economy studies. Global Environ Change 2005; 15(1): 33–43. doi: 10.1016/j.gloenvcha.2004.11.003

[17] Webb P, Coates J, Frongillo EA, Rogers BL, Swindale A, Bilinsky P. Measuring household food insecurity: why it’s so important and yet so difficult to do. J Nutr 2006; 136(5): 1404S–8S. doi: 10.1093/jn/136.5.1404S 16614437

[18] D’Souza A, Jolliffe D. Conflict, food price shocks, and food insecurity: the experience of Afghan households. Food Policy 2013; 42: 32–47. doi: 10.1016/j.foodpol.2013.06.007

[19] Ratcliffe C, McKernan SM, Zhang S. How much does the supplemental nutrition assistance program reduce food insecurity? Am J Agric Econ 2011; 93(4): 1082–98. doi: 10.1093/ajae/aar026 25197100 PMC4154696

[20] Hsiang S, Allen D, Annan-Phan S, Bell K, Bolliger I, Chong T, et al. The effect of large-scale anti-contagion policies on the COVID-19 pandemic. Nature 2020; 584(7820): 262–7. doi: 10.1038/s41586-020-2404-8 32512578

[21] WHO. WHO Director-General’s opening remarks at the media briefing on COVID-19 – 11 March 2020. Available from: https://www.who.int/dg/speeches/detail/who-director-general-s-opening-remarks-at-the-media-briefing-on-covid-19---11-march-2020 [cited 2 July 2020].

[22] Wuhan Bureau of Statistics. Statistical Bulletin of Wuhan’s National Economic and Social Development in 2019. Available from: http://tjj.wuhan.gov.cn/tjfw/tjgb/202004/t20200429_1191417.shtml [cited 4 July 2020].

[23] The Government of Wuhan. Wuhan overview: natural and geographical. Available from: http://www.wuhan.gov.cn/zjwh/whgk/ [cited 2 July 2020].

[24] Wu F, Zhao, S, Yu B, Chen, YM, Wang W, Song ZG, et al. A new coronavirus associated with human respiratory disease in China. Nature 2020; 579(7798): 265–9. doi: 10.1038/s41586-020-2008-3 32015508 PMC7094943

[25] Novel Coronavirus Epidemic Prevention and Control Headquarters in Wuhan. COVID-19 epidemic prevention and control interim measures in Wuhan. Available from: http://www.wuhan.gov.cn/zwgk/tzgg/202003/t20200316_972483.shtml [cited 4 July 2020].

[26] Novel Coronavirus Epidemic Prevention and Control Headquarters in Wuhan. Notice of Wuhan Novel coronavirus infected pneumonitis epidemic prevention and control headquarters (No. 12). Available from: http://www.gov.cn/xinwen/2020-02/11/content_5477104.htm [cited 2 July 2020].

[27] Wuhan Municipal Health Commission. Covid-19 epidemic situation in Wuhan (24 March 2020). Available from: http://wjw.wuhan.gov.cn/ztzl_28/fk/tzgg/202004/t20200430_1198541.shtml [cited 3 July 2020].

[28] Coates J, Swindale A, Bilinsky P. Household Food Insecurity Access Scale (HFIAS) for measurement of household food access: indicator guide (v. 3). Washington, DC: FHI 360/FANTA; 2007.

[29] Mohammadi F, Omidvar N, Houshiar-Rad A, Khoshfetrat MR, Abdollahi M, Mehrabi Y. Validity of an adapted Household Food Insecurity Access Scale in urban households in Iran. Public Health Nutr 2012; 15(1): 149–57. doi: 10.1017/S1368980011001376 21806860

[30] Knueppel D, Demment M, Kaiser L. Validation of the Household Food Insecurity Access Scale in rural Tanzania. Public Health Nutr 2010; 13(3): 360–7. doi: 10.1017/S1368980009991121 19706211

[31] Kim K, Kim MK, Shin YJ, Lee SS. Factors related to household food insecurity in the Republic of Korea. Public Health Nutr 2011; 14(6): 1080–7. doi: 10.1017/S1368980010003733 21299915

[32] Afridi F, Li SX, Ren YF. Social identity and inequality: the impact of China’s hukou system. J Public Econ 2015; 123: 17–29. doi: 10.1016/j.jpubeco.2014.12.011

[33] Zhong TY, Si ZZ, Crush J, Xu ZY, Huang XJ, Scott S, et al. The impact of proximity to wet markets and supermarkets on household dietary diversity in Nanjing City, China. Sustainability 2018; 10(5): 1465. doi: 10.3390/su10051465

[34] Tusiime HA, Renard R, Smets L. Food aid and household food security in a conflict situation: empirical evidence from Northern Uganda. Food Policy 2013; 43: 14–22. doi: 10.1016/j.foodpol.2013.07.005

[35] Navarro CAJ, Gironella GMP, Ignacio MSE. Association of household food security status with mother/caregiver-child pair’s nutritional status using HFIAS and FCS. Philipp J Sci 2018; 143(3): 493–501.

[36] Galanakis CM. The food systems in the era of the coronavirus (COVID-19) pandemic crisis. Foods 2020; 9(4): 523. doi: 10.3390/foods9040523 PMC7230343 32331259

[37] Zhong TY, Si ZZ. The state of Household Food Security in Nanjing, China (HUNGRY CITIES REPORT NO. 9). Available from: https://hungrycities.net/wp-content/uploads/2018/04/HCP9.pdf [cited 6 July 2020].

[38] Editorial, Food in a time of COVID-19. Nat Plants 2020; 6(5): 429. doi: 10.1038/s41477-020-0682-7 32415297

[39] Changjiang Daily, Changjiang Net. Supermarket is not open to the public, how to buy life supplies? See how these districts do. Available from: http://www.cjrbapp.cjn.cn/p/161699.html [cited 6 July 2020].

[40] Investment in Wuhan. The 5th Wuhan online food shopping strategy is coming! Purchase platform to add 8 more. Available from: https://mp.weixin.qq.com/s/efJPeyAIOrsnv_Kl0Iuqwg [cited 6 July 2020].

[41] Block P, Hoffman M, Raabe IJ, Dowd JB, Rahal C, Kashyap R, et al. Social network-based distancing strategies to flatten the COVID-19 curve in a post-lockdown world. Nat Hum Behav 2020; 4(6): 588–96. doi: 10.1038/s41562-020-0898-6 32499576

[42] Investment in Wuhan. Wuhan community group purchase food prices on the high level and few categories? Relevant parties responded to the six questions. Available from: https://mp.weixin.qq.com/s/7pbM9mM2CkpU9LNLx3T8Pw [cited 6 July 2020].

[43] Wolfson JA, Leung CW. Food insecurity and COVID-19: disparities in early effects for US adults. Nutrients 2020; 12(6): 1648. doi: 10.3390/nu12061648 PMC7352694 32498323

[44] Wuhan Public Security Bureau. Starting from Oct 1, Wuhan will allow non-residents to enter their homes with points. Available from: http://gaj.wuhan.gov.cn/jmzx/jqfb/202001/t20200108_653923.html [cited 8 July 2020].

[45] Wuhan Civil Affairs Bureau. Notice of Wuhan COVID-19 prevention and control command on further ensuring the livelihood of people in need affected by the epidemic. Available from: http://mzj.wuhan.gov.cn/zwgk_918/fdzdgk/ggfw/shjz/202005/t20200512_1312791.shtml [cited 8 July 2020].

[46] Glass RJ, Glass LM, Beyeler WE, Min HJ. Targeted social distancing design for pandemic influenza. Emerg Infect Dis 2006; 12(11): 1671–81. doi: 10.3201/eid1211.060255 17283616 PMC3372334

[47] Nicola M, Alsafi Z, Sohrabi C, Kerwan A, Al-Jabir A, Iosifidis C, et al. The socio-economic implications of the coronavirus pandemic (COVID-19): a review. Int J Surg 2020; 78: 185–93. doi: 10.1016/j.ijsu.2020.04.018 32305533 PMC7162753

[48] Wechat Pie (Weixin Pai). 2019 WeChat data report. Available from: https://mp.weixin.qq.com/s/vmhoiRzpBs7-JK_x2a7gZw [cited 11 November 2020].

